# Understanding the value of monocyte distribution width (MDW) in acutely ill medical patients presenting to the emergency department: a prospective single center evaluation

**DOI:** 10.1038/s41598-024-65883-8

**Published:** 2024-07-02

**Authors:** Marcela Kralovcova, Jiri Müller, Zdenka Hajsmanova, Pavla Sigutova, Lenka Bultasova, Jana Palatova, Martin Matejovic

**Affiliations:** 1 1st Department of Internal Medicine, Faculty of Medicine in Pilsen, Teaching Hospital, Charles University, Prague, alej Svobody 80, 323 00 Pilsen, Czech Republic; 2 Institute of Clinical Biochemistry and Hematology, Faculty of Medicine in Pilsen, Teaching Hospital, Charles University, Prague, alej Svobody 80, 323 00 Pilsen, Czech Republic

**Keywords:** Sepsis, Infection, Diagnostic biomarkers, Monocyte distribution width, Emergency department, Microbiology, Biomarkers, Diseases, Medical research

## Abstract

The monocyte distribution width (MDW) has emerged as a promising biomarker for accurate and early identification of patients with potentially life-threatening infections. Here we tested the diagnostic performance of MDW in adult patients requiring hospital admission for community-acquired infections and sepsis, evaluated sources of heterogeneity in the estimates of diagnostic accuracy, and assessed the meaning of MDW in a patient population presenting to the emergency department (ED) for acute non-infectious conditions. 1925 consecutive patients were categorized into three groups: non-infection (n = 1507), infection (n = 316), and sepsis/septic shock (n = 102). Diagnostic performance for infection or sepsis of MDW alone or in combination with components of SOFA was tested using AUC of ROC curves, sensitivity, and specificity. The relationship between MDW and different pathogens as well as the impact of non-infectious conditions on MDW values were explored. For the prediction of infection, the AUC/ROC of MDW (0.84) was nearly overlapping that of procalcitonin (0.83), and C-reactive protein (0.89). Statistical optimal cut-off value for MDW was 21 for predicting infection (sensitivity 73%, specificity 82%) and 22 for predicting sepsis (sensitivity 79%, specificity 83%). The best threshold to rule out infection was MDW ≤ 17 (NPV 96.9, 95% CI 88.3–100.0), and ≤ 18 (NPV 99.5, 95% CI 98.3–100.0) to rule out sepsis. The combination of MDW with markers of organ dysfunction (creatinine, bilirubin, platelets) substantially improved the AUC (0.96 (95% CI 0.94–0.97); specificity and sensitivity of 88% and 94%, respectively). In conclusion, MDW has a good diagnostic performance in diagnosing infection and sepsis in patients presenting in ED. Its use as an infection marker even increases when combined with other markers of organ dysfunction. Understanding the impact of interactions of non-infectious conditions and comorbidities on MDW and its diagnostic accuracy requires further elucidation.

## Introduction

Infections are one of the most common reasons for patients to seek medical care in the emergency department. The high burden of infections has been demonstrated in a large German study showing that more than one out of four patients admitted to the hospital were diagnosed with infection^1^. Despite major advances in medicine, early recognition of potentially life-threatening infections remains a fundamental challenge in clinical practice. The spectrum of symptoms is highly variable and non-specific. Early signs of sepsis are often vague or unusual and may be missed, misinterpreted or clouded by complex underlying disease conditions. Many serious non-infectious diseases may mimic sepsis. The failure to rule out an infection accurately can also lead to excessive and unnecessary use of antimicrobial therapy. Unfortunately, available laboratory tests for early and accurate diagnosis of serious infections have significant limitations^2^.

Recently, there has been a resurging interest in studying the diagnostic utility of various components of the complete blood cell count, which represents a first-level test in all acutely ill patients. Indeed, complete blood count hides a rich collection of useful information related to each blood cell^3^. Emerging data suggest that monocyte distribution width (MDW), a quantitative measure of variability in the volume of circulating monocytes, might improve early screening and diagnosis of acute serious infections, thus navigating clinical decision-making in combination with clinical findings^4–14^. The performance of MDW coupled with white blood cell count (WBC) has been reported to be equivalent to or outperform the accuracy of C-reactive protein (CRP) and procalcitonin (PCT) for the diagnosis of sepsis^15–20^. Especially in a subgroup of patients with a low pre-test sepsis probability score, in which no CRP or PCT had been ordered routinely^21^. Recently published systematic reviews and meta-analyses demonstrated promising diagnostic performance of MDW both for bacterial and viral sepsis, including COVID-19 and it has been suggested that MDW, due to its high sensitivity, could serve as a “rule-out” screening tool for sepsis in adult patients^22,23^. In addition, the above-mentioned metanalyses suggested that the overall diagnostic performance of MDW was comparable with that of PCT and CRP. Although encouraging, original studies included in recent meta-analyses have been performed in a heterogeneous clinical context, with inconsistent thresholds for MDW and variable outcome definitions. Thus, further studies are required to understand the role of MDW in the early detection of serious infections. With this background, the primary research objective of the study was to test the diagnostic performance of MDW in adult patients requiring hospital admission for community-acquired infections, sepsis and/or septic shock and to investigate sources of heterogeneity in the estimates of diagnostic accuracy. We also sought to evaluate the meaning of MDW biomarker in a large and unselected patient population presenting to the emergency department for acute non-infectious conditions.

## Material and methods

### Study design, population, and setting

We performed observational, exploratory, prospective, non-interventional cohort study in a high-volume academic center. The study was conducted between September 2019 and October 2020. The study was performed in accordance with Good Clinical Practice, as defined by the International Conference on Harmonization, the ethical principles underlying European Union Directive 2001/20/EC, and all applicable local requirements. The need for informed consent was waived given the non-interventional, no potential harm to subjects, and anonymous nature of the study. MDW results were unavailable to the physicians in charge and subjects were not managed based on the results of MDW.

The study population included all consecutive adults presenting to the Emergency department and subsequently admitted to the Department of Internal Medicine (medical ward or intensive care unit). For the final analysis, patients were subsequently assigned into one of three pre-defined groups based on the electronic medical records: (1) patients without clinically, radiologically, microbiologically and/or laboratory (negative PCT levels) proven infections; (2) patients with definitive admission diagnosis of bacterial infection (positive bacterial culture result, and/or clinical, radiological and laboratory findings, (3) patients with definitive admission diagnosis of sepsis and/or septic shock (defined according to Sepsis-3 criteria)^24^. The assignment to the respective groups was performed by analyzing hospital electronic medical records after completion of the study. The hospital electronic medical records were reviewed by two experienced clinicians (MK, JM). All patients were assessed by both investigators. Discrepancies were resolved by consulting a third investigator (MM). All diagnoses were made after consensus and continuous data integrity checks by independent investigators.

### Data collection

All clinical and paraclinical data were recorded in and extracted from a hospital electronic medical record (Medicalc4). Baseline demographic and clinical variables included age, sex, co-morbidities (cancer, diabetes, cirrhosis, chronic heart failure, coronary artery disease, chronic kidney disease, chronic obstructive pulmonary disease, immunosuppression), vital signs, qSOFA, SIRS, routine biochemistry and microbiological data, ward or intensive care admission, hospital length of stay and survival. Blood for the complete blood count with differential was obtained in a K3EDTA tube. The same blood sample was used for MDW measurement using UniCelDxH 900 analyzer (Beckman Coulter, Inc., Brea, CA). C-reactive protein (CRP) was determined in all patients (cobas c 702, Roche; Tina-quant, immunoturbidimetry, reference range 0–5 mg/l), while procalcitonin (PCT) was measured (cobas e 601, Roche; Elecsys BRAHMS, ECLIA, electrochemiluminescence) when clinically indicated.

### Study outcome and statistical analysis

The primary aim of this study was to evaluate the diagnostic utility of MDW value to detect an infection, sepsis and/or septic shock in the population of patients presenting to the emergency department and requiring admission to the hospital. We sought to determine the optimal cut-off, rule-in and rule-out value and then compare the MDW performance with other well established infection biomarkers. The secondary aim was to investigate the optimal statistical model combining MDW with other easily obtainable laboratory parameters such as infection biomarkers or SOFA score components.

General descriptive statistics for the study population were calculated. Continuous variables are presented as a median with IQR (interquartile range). Categorical variables are presented as numbers with percentages. Statistical differences between continuous variables were evaluated by the non-parametrical Kruskal–Wallis test or Wilcoxon test. To determine the optimal cut-off, rule-in and rule-out value for MDW and to compared the MDW performance with other biomarkers we performed multiple receiver operating characteristic (ROC) analysis, including the calculation of sensitivity, specificity, likelihood ratio and the AUC (Area Under the Curve). The best thresholds were searched by the Youden method from ROC curve analysis. ORs (odds ratios) were calculated for the optimal cut-off values. Results presented along with their 95% confidence intervals (CIs). The results were considered significant for p value less than 0.05. For the non-infectious group we defined the 95^th^ percentile for MDW and then compared MDW values between different groups using non parametrical one-way ANOVA on ranks. Spearman’s rank correlation was computed to assess the relationship between MDW and qSOFA score.

A multivariate logistic regression analysis was used to detect statistically significant predictors of sepsis development. Those variables were then subjected to a final logistic regression model. The ROC analysis of this model, including sensitivity and specificity, was then compared to the best standard biomarker (CRP).

We used SAS (SAS Institute Inc., Cary, NC, USA) and Statistica 12 (StatSoft^®^, TIBCO Software Inc., USA) for the statistical analysis.

### Ethics approval

This study’s protocol was approved by the ethics committee of Faculty hospital Pilsen, Czech Republic (approval number: 393/2020).

## Results

**Figure 1:**
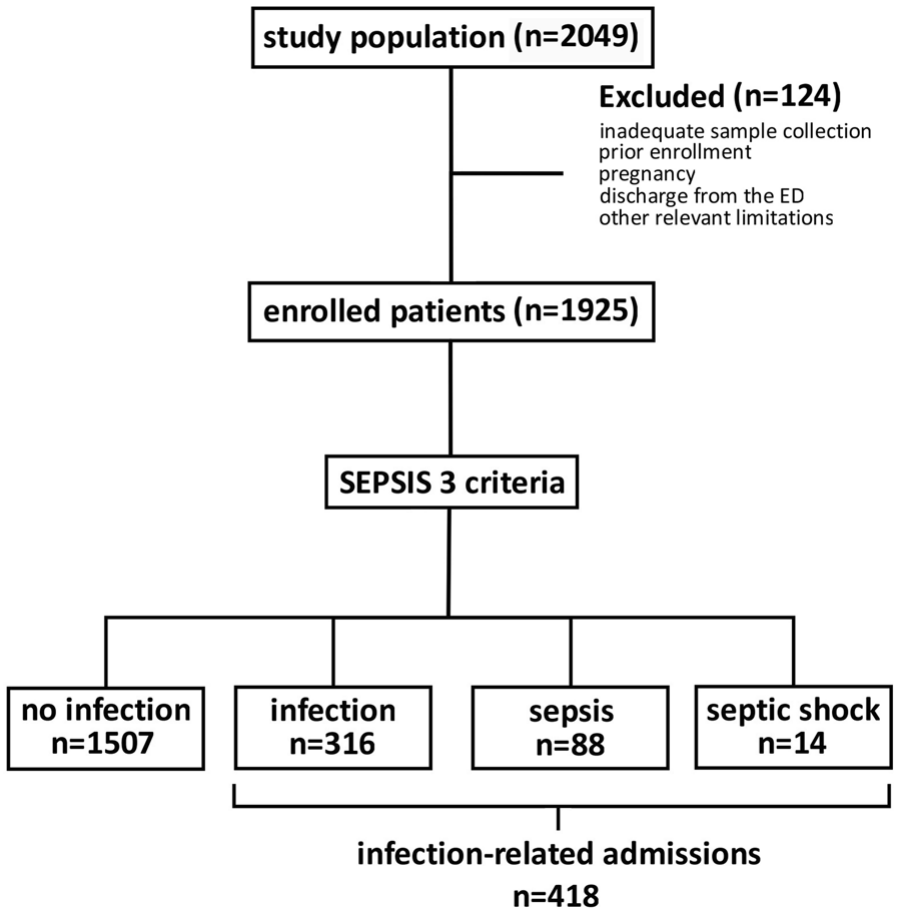
Flowchart describing enrollment and exclusion of patients.

A total of 2049 adult patients presenting to the Emergency department and subsequently admitted to the hospital were assessed for eligibility during the reference timeframe. Of these patients, 124 patients were excluded due to inadequate sample collection (inaccurate MDW measurement), prior enrollment in the study, pregnancy, discharge from the ED or due to other relevant limitations (e.g. absence of other biomarkers such as CRP). Thus, 1925 patients were enrolled, of whom 418 were admitted with infection-related causes. The flowchart of the enrollment process is shown in Fig. 1.

All those patients had complete blood count differential including MDW, PCT, CRP, coagulation (aPTT–activated Partial Thromboplastin Time, PT–Prothrombin Time) and biochemistry needed for SOFA score calculation (creatinine, bilirubin, lactate) ordered upon presentation to the emergency department. Group 1 included patients without an infection (n = 1507; 78.3%); group 2 included patients that developed an infection but did not meet the criteria for sepsis (n = 316; 16.4%) and group 3 included patients with sepsis and/or septic shock, defined by a vasopressor requirement to maintain a mean arterial pressure of 65 mmHg or greater and serum lactate level greater than 2 mmol/L in the absence of hypovolemia (n = 102, 5.3%). Characteristics of patients at baseline and preexisting medical conditions likely to predispose for infection are provided in Table 1. Baseline blood count differential including MDW, CRP, PCT and biochemistry values are provided in Table 2.

**Table 1:** Characteristics of the patients at baseline.

Characteristic	Group 1–no infection (n = 1507)	Group 2–infection (n = 316)	Group 3–sepsis and septic shock (n = 102)
Age–years	59 (41;73)	68 (46;78)	75 (69;84)
Sex–no. (%)			
Male	682 (45.3)	129 (40.8)	57 (55.9)
Female	825 (54.7)	187 (59.2)	45 (44.1)
Coexisting condition–no. (%)			
COPD	77 (5.1)	24 (7.6)	11 (10.8)
Diabetes	276 (18.3)	87 (27.5)	39 (38.2)
CKD	114 (7.6)	52 (16.5)	42 (41.2)
Immunosuppression	35 (2.3)	24 (7.6)	13 (12.8)
Chronic heart failure	193 (12.8)	60 (19)	32 (31.4)
Coronary artery disease	220 (14.6)	64 (20.3)	25 (24.5)
Cirrhosis	14 (0.9)	0 (0)	7 (6.9)
Cancer	126 (8.4)	52 (16.5)	30 (29.4)
qSOFA score–no. (%)			
0 and 1	1410 (94.2)	272 (86.3)	51 (50.5)
2	81 (5.4)	39 (12.4)	44 (43.6)
3	6 (0.4)	4 (1.3)	6 (5.9)

*IQR* interquartile range, *COPD* chronic obstructive pulmonary disease, *CKD* chronic kidney disease, *qSOFA* quick sequential organ failure assessment.

**Table 2:** Laboratory results of monitored parameters and parameters of SOFA score.

Characteristic	Group 1 no infection (n = 1507)	Group 2 infection (n = 316)	Group 3 sepsis (n = 102)	p value*
Blood count differential				
WBC (× 10^9^/L)	8.2 (6.7–10)	10.2 (7.8–13)	11.7 (8.5–15.9)	< 0.001
Neutrophils (%)	69 (61–77)	81 (65–87)	84 (78–89)	0.006
Lymphocytes (%)	19 (14–26)	11 (6–20)	9 (5–13)	< 0.001
NLR	3.5 (2.9–5.5)	8 (2.9–15.2)	9.5 (5.7–14.0)	0.004
MDW (U)	19 (17–20)	22 (20–25)	24 (22–27)	< 0.001
Platelets (× 10^9^/L)	239 (197–287)	239 (197–298)	193 (133–244)	< 0.001
Biochemistry				
C-reactive protein (mg/L)	3 (1–8)	39 (15–91)	116 (46–206)	< 0.001
Procalcitonin (μg/L)	0.05 (0.02–0.07)	0.12 (0.07–0.26)	0.87 (0.23–2.49)	< 0.001
Creatinine (μmol/L)	79 (67–96)	82 (68–103)	135 (99–211)	< 0.001
Bilirubin (μmol/L)	8 (6–12)	11 (7–15)	24 (11–36)	< 0.001
Lactate (mmol/L)	1.6 (1.2–2.2)	1.6 (1.2–1.7)	1.4 (1–2.9)	0.58
Coagulation				
aPTT (seconds)	30 (27–33)	31 (28–36)	32 (28–38)	0.002
PT (seconds)	12 (11–13)	13 (12–15)	15 (13–17)	< 0.001

*Apart from lactate every parameter reached statistical significance, both in inter-group comparison and sepsis/septic shock to other groups comparison. Displayed p values stands for sepsis/septic shock group.

*WBC* white blood count, *NLR* neutsrophil-to-lymphocyte ratio, *MDW* monocyte distribution width, *aPTT* activated partial thromboplastin time, *PT* prothrombin time.

### MDW for detection of infection and sepsis

**Figure 2:**
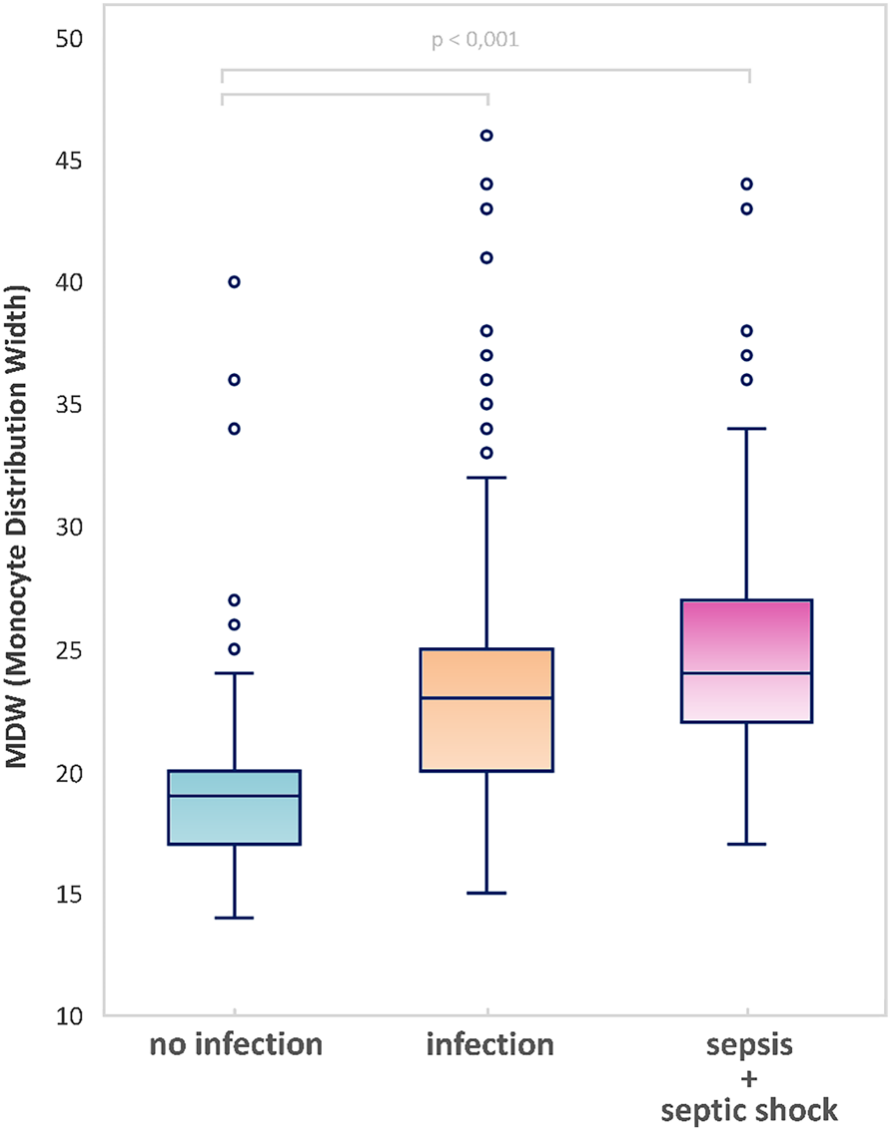
Box plot showing significantly higher MDW values for patients with an infection (with or without sepsis and/or septic shock) or with sepsis and/or septic shock according to Sepsis-3 cristeria compared to the group of patients without an infection.

MDW was significantly higher in patients admitted with infection (without or with sepsis and/or septic shock) compared to those without infection [23(20–25) vs. 19(17–20), p < 0.001]. Similarly, patients with sepsis and septic shock according to SEPSIS-3 criteria had statistically higher MDW than subjects without infection [24 (22–27) vs.19 (17–20), p < 0.001], as shown in Fig. 2.

For the prediction of an infection, the AUC (Area Under the Curve) of MDW, obtained by the ROC curve analysis, was 0.839 (95% CI 0.82–0.86) which was the second highest after the AUC of CRP [0.894 (95% CI 0.88–0.91)], followed by that of PCT [0.826 (95% CI 0.77–0.88)] and WBC [0.685 (95% CI 0.65–0.72)]. Statistically, the optimal cut-off value for predicting infection was 20.66 [OR = 12.38 (95% CI 9.62–15.94)] with a specificity of 82.3% (95% CI 80.4–84.2) and a sensitivity of 72.73% (95% CI 68.5–77.0), while the LR + (positive likelihood ratio) and LR- (negative likelihood ratio) were 4.1 and 0.33 respectively. The best threshold to rule out infection was MDW ≤ 17 (NPV 96.9, 95% CI 88.3–100.0), to rule in optimal value was MDW > 28 (PPV 91.1, 95% CI 84.7–97.4).

**Figure 3:**
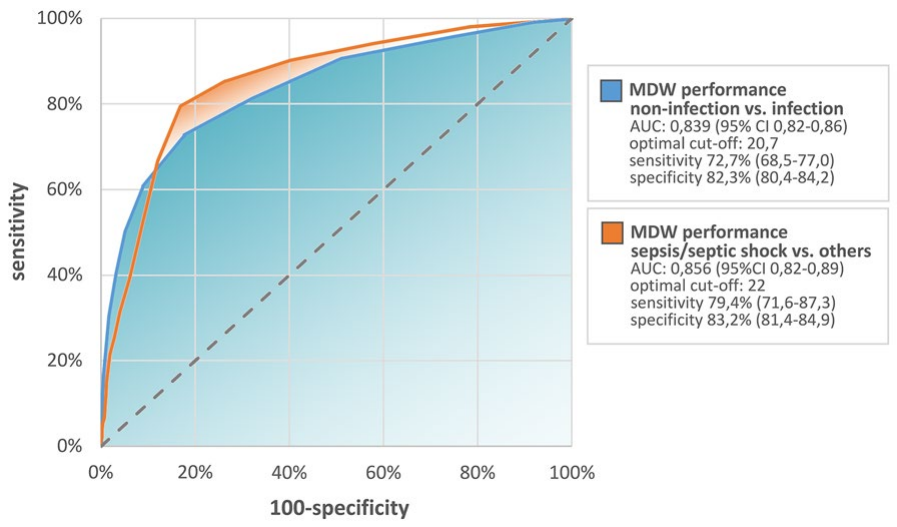
ROC curves analysis comparison for MDW in prediction of sepsis and or septic shock (blue) or infection (orange) upon presentation to the emergency department.

The AUC/ROC analysis for MDW performance in detection of sepsis and septic shock according to Sepsis-3 criteria was 0.856 (95% CI 0.82–0.89), slightly below CRP and PCT [0.915 (95% CI 0.89–0.94); 0.872 (95% CI 0.83–0.92) respectively]. Optimal cut-off for prediction of sepsis and septic shock was 22 [OR = 19.05(95% CI 11.61–31.26)] with a specificity of 83.16% (95% CI 81.4–84.9) and sensitivity of 79.41% (95% CI 71.6–87.3) with LR + 4.72 and LR- 0.25]. Based on our data, the most precise MDW value to rule in sepsis was MDW > 43 (PPV 66.7, 95% CI 20.47–100) and MDW ≤ 18 (NPV 99.5, 95% CI 98.3–100) to rule out sepsis. ROC curves for infection and sepsis detection are showed in Fig. 3.

### Non-infectious causes for MDW elevation

**Figure 4:**
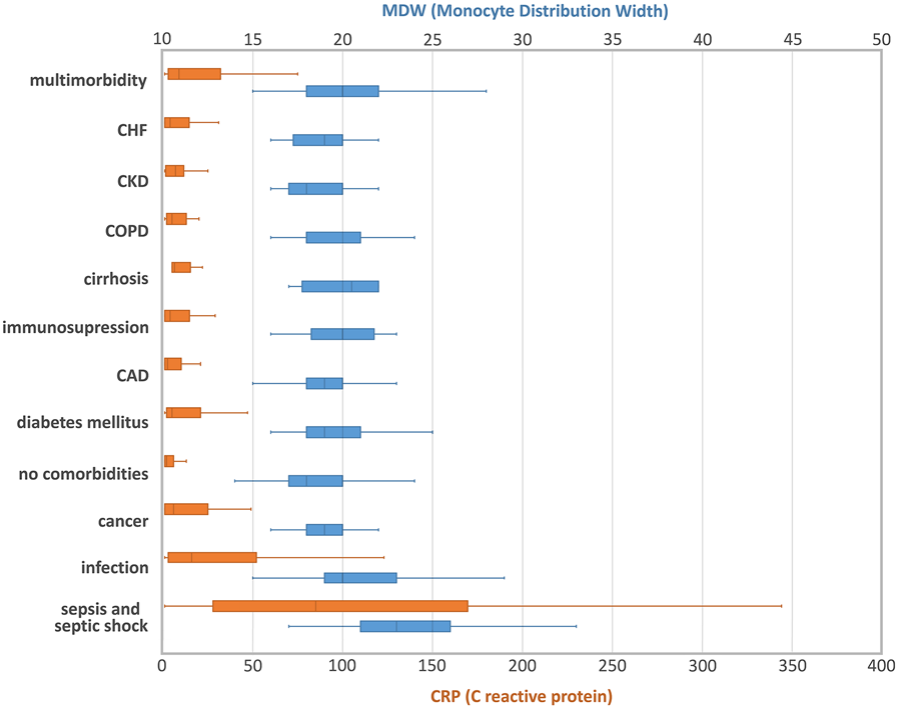
Comparison of MDW and CRP in non-infection, infection and sepsis/septic shock groups of patients. Medians with interquartile ranges. *CAD* coronary artery disease, *CHF* chronic heart failure, *CKD* chronic kidney disease, *COPD* chronic obstructive pulmonary disease, *DM* diabetes mellitus.

The distributions of non-infectious causes of MDW and CRP across various diagnostic groupings are shown in Fig. 4.

In the non-infectious group diabetes mellitus and multimorbidity were associated with higher MDW values than that of patients without comorbidities. Multimorbidity was defined as at least two unrelated chronic conditions from other groups, with diabetes being the most common (69%). Other groups did not reach statistical significance, although both patient with cirrhosis and immune-suppression had higher or identical median values (Table 3).

**Table 3:** Comparison of non-infective causes for MDW elevation.

Group	MDW (IQR)	p value*
Patients with comorbidities		
Cancer	19 (18–20)	
Diabetes mellitus	19 (18–21)	< 0.001
CHF	19 (18–20)	
CAD	19 (18–20)	
COPD	18 (18–21)	
Multimorbidity	20 (18–22)	< 0.001
CKD	18 (17–20)	
Immune-suppression	20 (19–21)	
Cirrhosis	21 (19–22)	
Patients without comorbidities	18 (17–20)	

*p value for significant difference between comorbidities and no comorbidities.

The median value of MDW in non-infectious group was 19 (17–20) with maximum of 40 and 95th percentile 23. Thus, we selected all patients above 95th percentile for MDW to evaluate the cause of the elevation. Total of 49 patients crossed the threshold, of which 30 had significant comorbidities. (Table S1, Supplementary Appendix) For the purpose of the risk factor analysis in a limited sample setting we counted each comorbidity separately in multimorbid patients. The median and mean values with IQR are provided in Table 4. Interestingly, 50% of the patients with comorbidities and high MDW without an infection had active cancer, which is a significant proportion considering only 10.8% of patients with comorbidities from the whole study population had cancer and the percentage was even lower in the non-infective group (8.4%). The most common type of cancer in this group were hematologic malignancies (n = 7; 46.7%) followed by adenocarcinoma of pancreas (n = 3; 20%). Moreso, four patients with significant MDW elevation without an infection or comorbidities had cancer diagnosed on admission. No significant difference in MDW values was found in-between patients grouped by comorbidities.

**Table 4:** Non-infective causes of significant MDW elevation above 95th percentile (MDW value 23).

Group	Number (%)	MDW (IQR)
All non-infective patients	49	25 (24–26)
Patients with comorbidities	30	24 (24–25)
Cancer	15 (50)	25 (24–26)
Diabetes mellitus	12 (40)	25 (25–26)
CHF	9 (30)	25 (24–26)
CAD	8 (27)	25 (24–25)
COPD	8 (27)	24 (24–25)
CKD	5 (17)	25 (24–26)
Immune-suppression	5 (17)	24 (24–25)
Cirrhosis	2 (7)	25 (25–26)
Patients without comorbidities	19	25 (24–27)

*CHF* chronic heart failure, *CAD* coronary artery disease, *COPD* chronic obstructive pulmonary disease, *CKD* chronic kidney disease.

### MDW in combination with qSOFA and other biomarkers of infection

**Figure 5:**
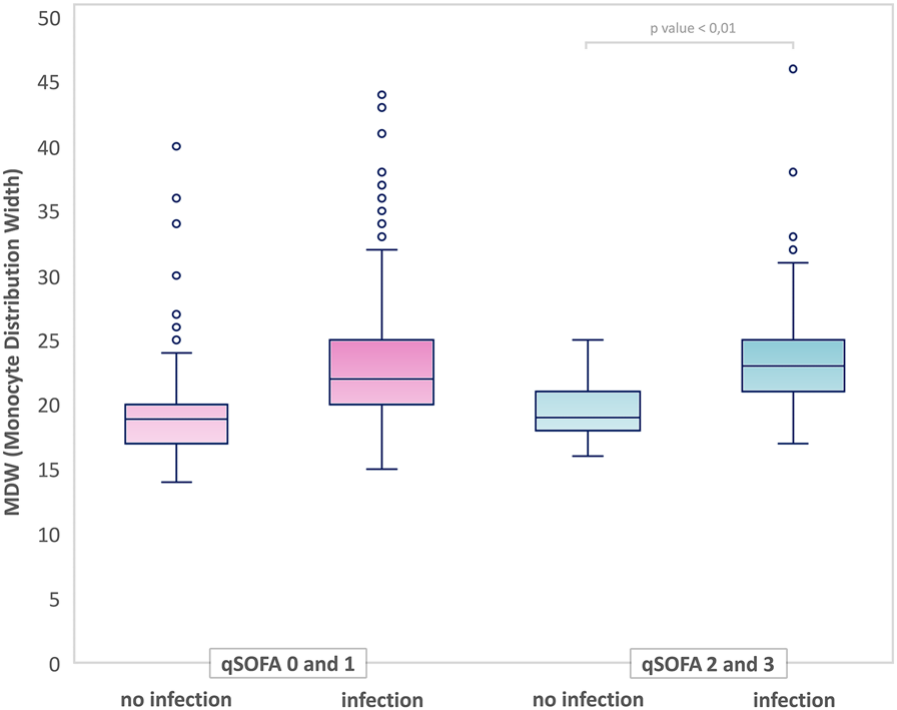
Infection and no-infection groups comparison based on qSOFA score.

There was a weak positive correlation between MDW and qSOFA in the non-infective group (r(1500) = 0.09, p < 0.001) and no significant correlation in the infective group based on Spearman’s rank calculation. However, in contrast to qSOFA score elevation (2 and 3 points) from non-infectious etiology, we observed a higher MDW values in patients with positive qSOFA and infection [MDW 19 (18–21) vs. 23 (21–25) respectively; p value < 0.001) (Fig. 5).

**Figure 6:**
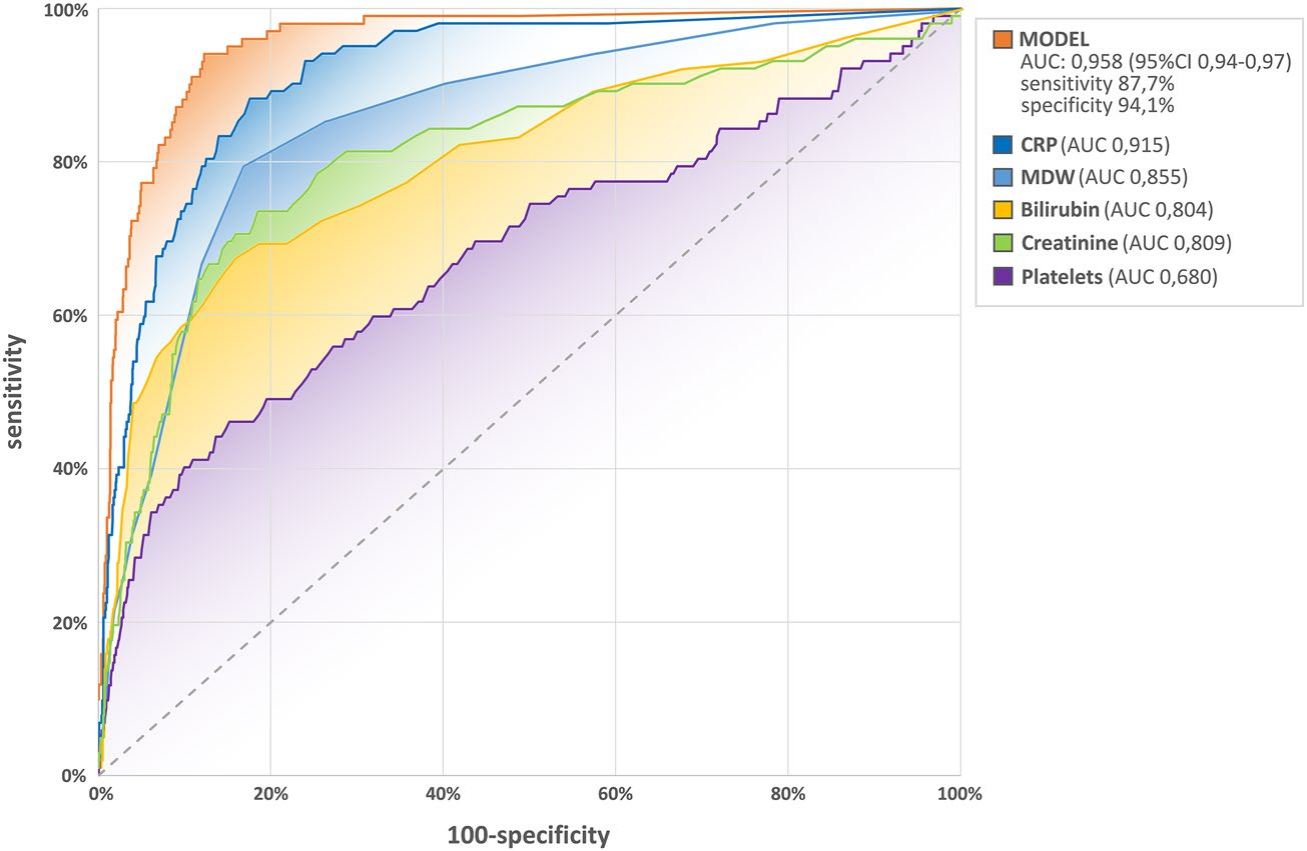
Diagnostic performance of MDW combined with SOFA score parameters compared with MDW alone. ROC curves comparisons with AUC. *CRP* C-reactive protein, *MDW* monocyte distribution width.

In the multivariate logistic regression analysis MDW (OR: 1.083; 95% CI 1.014–1.159; p < 0.02) as well as CRP (OR: 1.017; 95% CI 1.013–1.021; p < 0.001), platelets (OR: 0.991; 95% CI 0.987–0.995; p < 0.001), bilirubin (OR: 1.026; 95% CI 1.018–1.034; p < 0.001) and creatinine (OR: 1.007; 95% CI 1.005–1.009; p < 0.001) were found independently associated with sepsis development (Table 5). We evaluated the diagnostic performance of MDW combined with those biomarkers of infection and SOFA score parameters. The AUC/ROC analysis of this model [0.958 (95% CI 0.94–0.97)] yielded better results than the best biomarker alone–CRP (p < 0.001), with specificity and sensitivity of 88% and 94%, respectively. ROC curves comparisons are showed in Fig. 6.

**Table 5:** Results of the multivariate logistic regression analysis.

Predictors	Regression coefficient (β)	DF (degree of freedom)	SE (Standard Error)	Wald Chi-Square test	aOR (adjusted Odds Ratio)	95% CI for aOR	p value
Intercept	− 5.030	1	0.868	33.497	0.007	0.002–0.035	< 0.001
MDW	0.081	1	0.034	5.797	1.084	1.014–1.159	0.02
CRP	0.017	1	0.002	75.658	1.017	1.013–1.021	< 0.001
Platelets	-0.009	1	0.002	24.802	0.991	0.987–0.995	< 0.001
Bilirubin	0.026	1	0.004	33.597	1.026	1.018–1.034	< 0.001
Creatinine	0.007	1	0.001	56.321	1.007	1.005–1.009	< 0.001

*MDW* monocyte distribution width. *CRP* C-reactive protein.

### Effect of different causative pathogens on MDW Value

**Figure 7:**
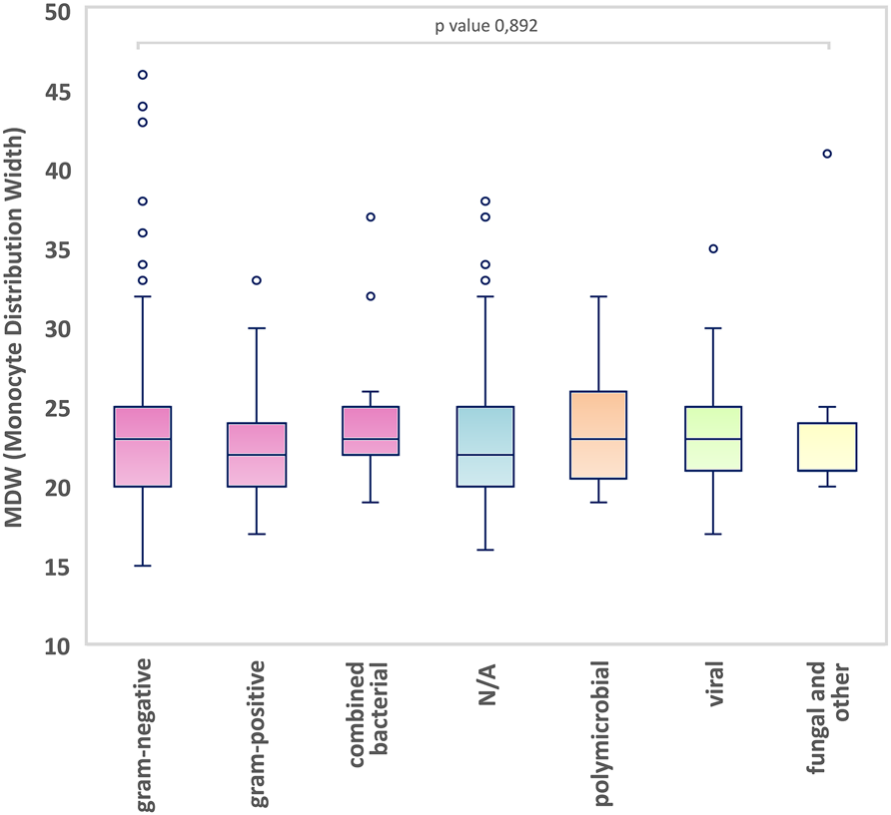
Boxplot of MDW values according to causative infective pathogen. No statistical significance was found in intergroup analysis; *N/A* no causative pathogen isolated.

In the infectious group (n = 418) the documented causative pathogens were Gram-negative bacteria (138 cases; 33%); Gram-positive bacteria (49 cases; 12%); combined Gram-positive and Gram-negative infection (23 cases; 6%); viral infection (29 cases; 7%); fungal and other types of infections (6 cases; 1.4%) and polymicrobial infection (7 cases; 1.7%). In 166 cases (40%) no definitive pathogen was identified. No statistically significant difference was found between groups (p value 0,892), as depicted in Fig. 7.

## Discussion

This study confirmed that MDW has a high diagnostic value in medical patients presenting in the emergency department with suspected infections requiring admission to the hospital. We now demonstrate for the first time that MDW significantly augments detection of potentially life-threatening infections when used in combination with routine biomarkers of organ function. Additionally, our data suggest that the MDW-enhanced-qSOFA assessment, which combines MDW and qSOFA, modulates the probability of serious infections predicted from qSOFA positivity. Finally, we provide the first evidence related to the interaction between comorbidities and MDW values. Collectively, our findings validate and further extend the observations made in previous studies using MDW for the diagnosis of infection and/or sepsis.

Our results indicate that MDW, with a sensitivity of 73%, specificity of 82%, and an AUC of 0.84 possesses good accuracy in discriminating infections from non-infectious conditions and display a good performance in sepsis prediction with a sensitivity of 79%, specificity 83% and an AUC of 0.86. Those findings are consistent with the previous reports^4–14^. The cut-off value 22 for sepsis prediction, as determined in our study, was similar to that reported by Jo et al.^6^, Polili et al.^13,17^ and Hausfater et al.^19^, yet slightly higher than values obtained in other studies^4,5,7,10–12,14–17,19^. This could be explained by the type of anticoagulant used for blood samples. K3-EDTA anticoagulated blood samples are consistently associated with higher MDW values than those with K2-EDTA, thus different cut-off is recommended. Positive and negative LRs were 4.1 and 0.33, respectively, further reinforcing the reliability and precision of low MDW as a biomarker in “ruling out” infection. The AUC/ROC analysis of MDW for infection and sepsis (0.84 and 0.86 respectively) was found to be similar to that of CRP (0.89 and 0.92 respectively) and PCT (0.83 and 0.87 respectively), suggesting comparable performance in early detection of infection and sepsis, but avoiding complexity to the laboratory work required.

Sepsis is an extremely heterogeneous syndrome with variability in pathogen, source of infection, comorbidities and patient's genetic make-up. Given this heterogeneity, a single biomarker may not be able to sufficiently reflect the underlying complexity. This has kindled interest in the use of biomarker combinations. Indeed, algorithms that combine MDW with other biomarkers have shown promise in more robust identification of patients with sepsis in the emergency department when compared to MDW alone^4,10,11,14,19,21^. For the first time, in our study, we used completely different approach by combining MDW as an indicator of activated innate immune system with markers of acute organ dysfunction, both reflecting pathophysiologically distinct processes. A key finding in the multivariable analysis was that a new model consisting of MDW and three biomarkers of organ injury/dysfunction (creatinine, bilirubin, platelets) yielded an AUC 0.96, with an impressive discriminating sensitivity and specificity values reaching 88% and 94%, respectively, indicating that this model could serve as a sensitive indicator for the early detection of sepsis.

We also sought to determine, whether MDW may aid in distinction between infectious and non-infectious causes of positive qSOFA, which has been proposed by sepsis-3 criteria as a tool to identify patients with suspected infection at risk for poor outcomes^25^. However, recent study showed that only one in three patients with ≥ 2 qSOFA criteria on admission has suspected infection^26^. In our cohort, 87 of patients were positive for qSOFA, despite no evidence of infection. In these patients, the use MDW seems valuable, easy-to-obtain biomarker in ruling out the suspicion of serious infection within a very short turnaround time. The usefulness of the combination of MDW and qSOFA in augmenting diagnostic accuracy for early sepsis detection in ED has been supported by two recent studies^5,17^.

Because different pathogens might trigger distinct pathogen-specific immune response and signaling, we also analyzed whether the modulation of MWD is influenced by specific pathogens. Interestingly, with the exception of two studies^6,7^, the majority of reports evaluated the utility of MDW as a sepsis biomarker without considering the causing pathogen. In line with the two above-mentioned studies, our analysis did not suggest that specific groups of pathogens (Gram-negative bacteria, Gram-positive bacteria; combined Gram-positive and Gram-negative infection; viral; fungal and other types of infections) have a relevant influence on MDW values. This makes MDW suitable as a screening biomarker for any severe infection, independent of the causative pathogen^8^.

Understanding the impact of non-infectious conditions on MDW and its diagnostic accuracy is of great clinical importance. Unfortunately, there is very limited knowledge concerning those interactions. We have shown that MDW is statistically elevated in patients with infection relative to those with noninfectious conditions. Nevertheless, our study also revealed the new finding that comorbidities, in particular multimorbidity, diabetes, cirrhosis and immune-suppression could modify the MDW pattern. Admittedly, the biological interpretation of associations between various comorbidities and MDW is not straightforward from our data. We can only speculate that specific comorbidities and overall multimorbidity are associated with an enhanced systemic inflammatory response that may be implicated in the change in the volume of circulating monocytes^27–29^. In addition, we have identified a minor subset of 49 patients with no proven infection, but presenting with markedly elevated MDW (maximum of 40 and 95th percentile 23). Interestingly, 50% of these patients had active cancer. Although we cannot account for all potential biases and confounding factors that can influence the interpretation of strikingly high MDW values, we believe that gaining this information could be of relevance to clinicians and a signal for more intensive research required to identify the mechanistic and causal relationships between non-infectious conditions and MDW.

Although this analysis contributes to the current knowledge about how MDW might support clinical judgement in patients presenting to the ED, it is not without limitations. The study required an analysis of clinical data extracted from hospital electronic medical records. Despite the fact that the assignment to the pre-specified groups was performed by two independent experts, misclassification of final diagnosis cannot be completely eliminated in some cases. All available clinical, microbiological, and radiological evidence was used as the “gold standard” for infection in our study. Perhaps using a more detailed infection criteria tool such as Linder-Mellhammar might yield higher accuracy for infection diagnoses in equivocal cases^30^. However, satisfactory correction for the bias introduced by an imperfect gold diagnostic standard of infection or sepsis does not exist. Furthermore, on an individual level, we found a large overlap in biomarker values between infectious and non-infectious conditions. Hence, larger external validation of derived findings is required to tease out the reproducibility and specificity in different patient groups and the role of the sampling window in the value of the measurements. In this context, we were confined to admission samples only, and, therefore, we were unable to assess the value of the serial MDW monitoring in evaluating clinical deterioration, response to therapy and other clinically meaningful outcomes. It is possible that serial biomarker measurement may have yielded different information and a different predictive ability. On the other hand, the strengths of this study include fairly large cohort of consecutive ED patients, including those without a clinical suspicion of infection. Such an approach of not utilizing healthy subject as a comparator is clearly advantageous, since it allowed us to test MDW performance in “real-world” scenario in ED, and, thereby, enabling to understand various factors that can influence the interpretation of MDW within individual patient context.

## Conclusion

In conclusion, taken together with previously published results, this study supports the role of MDW in point-of-care testing in the evaluation of patients presenting to emergency departments and acute settings with suspected infections, in particular as part of a decision tree in combination with other routinely available data. A deeper understanding of the interactions between MDW, co-morbidities and non-infectious conditions is warranted to better inform clinical decision making.

### Supplementary Information

Supplementary Information.

## Data Availability

The data that support the findings of this study will be available from the first author upon reasonable request (email: matejovic@fnplzen.cz). Supporting data will be made available to Editorial Board Members and referees at the time of submission for the purposes of evaluating the manuscript and directly upon request to any reader on and after the publication date. Supporting datasets will be made available as Supplementary Information files that will be freely accessible on the journal´s website upon publication.
