# Supplementary material for: Understanding the value of monocyte distribution width (MDW) in acutely ill medical patients presenting to the emergency department: a prospective single center evaluation
Source: Sci Rep. 2024 Jul 2;14:15255. doi: 10.1038/s41598-024-65883-8 (PMC11219845; doi:10.1038/s41598-024-65883-8)
Supplement: Supplementary file 1 — Supplementary Information. [file 41598_2024_65883_MOESM1_ESM.docx]

Supplementary Appendix

**Supplement to:** Understanding the value of monocyte distribution width (MDW) in acutely ill patients presenting to the emergency department: a prospective single center evaluation

This appendix has been provided by the authors to give readers additional information.

Definition of infection

In patients for whom the diagnosis of infection was unclear because of equivocal clinical, laboratory/radiological findings, or suspected false-negative or false-positive cultures (e.g. single cultures of coagulase negative cocci), “unlikely” infection was considered where clinicians thought there is a low probability of infection, did not consider antimicrobial treatment and/or source control. A probable infection was considered where clinicians thought there is a high probability of infection and the patient´s clinical condition required antimicrobial and/or source control. All diagnoses were made after consensus and continuous data integrity checks by independent investigators.

Experience of assessors:

MM: Level 2 (highest degree) board certified in internal medicine

Level 2 board certified in intensive care medicine

PhD in internal medicine and critical care

MK: Level 2 board certified in internal medicine, PhD student

JM: Level 1 board certified in internal medicine, 2 years experience in critical care, PhD student

**Table S1. Characteristics and admitting diagnosis of non-infectious patients with a significant MDW elevation (over 95th percentile).**

| **gender** | **age** | **admitting diagnosis** | **MDW** | **CRP** | **qSOFA** | **comorbidities** |
| --- | --- | --- | --- | --- | --- | --- |
| M | 75 | Anemia  cancer (lung) | 40 | 21 | 0-1 | N/A |
| F | 31 | severe allergic reaction (antibiotics) | 36 | 17 | 0-1 | N/A |
| F | 33 | deep vein thrombosis | 34 | 26 | 0-1 | N/A |
| F | 25 | arrhytmia (supraventricular tachycardia) | 30 | 8 | 0-1 | N/A |
| F | 27 | severe allergic reaction (antibiotics) | 27 | 7 | 0-1 | N/A |
| F | 78 | Hypocalcemia | 26 | 36 | 0-1 | N/A |
| F | 70 | seronegative rheumatoid arthritis | 26 | 87 | 0-1 | N/A |
| F | 84 | cancer (colorectal)  deep vein thrombosis  arrhytmia (atrial fibrillation)  heart failure | 25 | 38 | 0-1 | N/A |
| F | 49 | laceration of colon after colonoscopy | 25 | 32 | 0-1 | N/A |
| F | 50 | arrhytmia (supraventricular tachycardia) | 25 | 1 | 0-1 | N/A |
| F | 36 | severe allergic reaction (unknown trigger) | 25 | 15 | 0-1 | N/A |
| F | 60 | gastroenteritis | 24 | 3 | 0-1 | N/A |
| F | 69 | cancer (urinary bladder)  critical limb ischemia | 24 | 25 | 0-1 | N/A |
| F | 21 | upper gastrointestinal bleeding | 24 | 59 | 0-1 | N/A |
| F | 82 | atrial fibrillation  heart failure | 24 | 9 | 0-1 | N/A |
| F | 55 | syncope (vasovagal) | 24 | 2 | 0-1 | N/A |
| F | 84 | pulmonary embolism  cancer (lung) | 24 | 1 | 0-1 | N/A |
| M | 46 | renal insuficiency due to food poisoning | 24 | 182 | 0-1 | N/A |
| F | 72 | seronegative rheumatoid arthritis | 24 | 156 | 0-1 | N/A |
| F | 86 | acute myeloid leukemia | 40 | 19 | 0-1 | CHF |
| M | 67 | laceration of oesophagus after endoscopy | 27 | 185 | 0-1 | DM, CHF, CKD, COPD |
| F | 75 | severe hypertension | 27 | 3 | 0-1 | cancer |
| F | 36 | severe allergic reaction (antibiotics) | 26 | 21 | 0-1 | DM, CHF, CAD |
| M | 68 | pulmonary embolism  cancer (pancreas) | 26 | 62 | 0-1 | DM, COPD, cancer |
| M | 76 | atrial fibrillation  heart failure  myelodysplastic syndrome | 26 | 12 | 0-1 | CKD, CAD, cirrhosis, cancer |
| M | 54 | ulcerative colitis | 26 | 15 | 0-1 | DM, immunosuppression |
| M | 31 | diabetic ketoacidosis | 25 | 19 | 0-1 | DM |
| M | 69 | heart failure | 25 | 17 | 2-3 | DM, CHF, CKD |
| F | 83 | abdominal and back pain (neuralgia) | 25 | 1 | 0-1 | CAD, immunosupression, cancer |
| F | 79 | abdominal pain (abdominal angina) | 25 | 26 | 0-1 | DM, CHF, CAD, COPD |
| F | 68 | deep vein thrombosis  cancer (pancreas) | 25 | 2 | 2-3 | DM, cancer |
| F | 73 | cancer (colorectal, lung) | 25 | 8 | 2-3 | DM, cancer |
| M | 67 | arrhytmia (supraventricular tachycardia) | 25 | 21 | 0-1 | CAD, cancer |
| F | 64 | cancer (pancreas) | 24 | 33 | 0-1 | cancer |
| F | 59 | anemia | 24 | 1 | 0-1 | COPD, cirrhosis |
| M | 37 | diabetic ketoacidosis | 24 | 7 | 0-1 | DM |
| M | 63 | anemia | 24 | 2 | 0-1 | CAD |
| F | 75 | severe hypertension | 24 | 1 | 0-1 | cancer |
| F | 82 | COPD exacerbation | 24 | 10 | 0-1 | COPD |
| F | 71 | deep vein thrombosis  pulmonary embolism | 24 | 9 | 0-1 | immunosupression, cancer |
| M | 86 | renal insuficiency | 24 | 8 | 2-3 | CHF, CKD, CAD, COPD |
| F | 82 | COPD exacerbation | 24 | 16 | 2-3 | DM, CHF, COPD |
| M | 80 | deep vein thrombosis  pulmonary embolism | 24 | 41 | 0-1 | immunosupression, cancer |
| M | 78 | cancer (melanoma) | 24 | 29 | 0-1 | immunosupression, cancer |
| M | 86 | encephalopathy (toxic, due to medication) | 24 | 16 | 0-1 | CHF, CAD, COPD |
| M | 59 | encephalopathy (toxic, due to medication) | 24 | 6 | 0-1 | DM |
| F | 64 | deep vein thrombosis  pulmonary embolism | 24 | 215 | 0-1 | cancer |
| M | 20 | syncope (orthostatic) | 24 | 1 | 0-1 | CHF |
| M | 85 | upper gastrointestinal bleeding | 24 | 41 | 0-1 | CKD, cancer |

**Notes:** DM – diabetes mellitus, CHF – chronic heart failure, CAD – coronary artery disease, CKD – chronic kidney disease, COPD – chronic obstructive pulmonary disease, M – male, F – female
